# Associations between alteration in plant phenology and hay fever prevalence among US adults: Implication for changing climate

**DOI:** 10.1371/journal.pone.0212010

**Published:** 2019-03-28

**Authors:** Amir Sapkota, Raghu Murtugudde, Frank C. Curriero, Crystal R. Upperman, Lewis Ziska, Chengsheng Jiang

**Affiliations:** 1 Maryland Institute for Applied Environmental Health, School of Public Health, University of Maryland, College Park, MD, United States of America; 2 Department of Atmospheric and Oceanic Science, University of Maryland, College Park, MD, United States of America; 3 Department of Epidemiology, Bloomberg School of Public Health, Johns Hopkins University, Baltimore, MD, United States of America; 4 Crop Systems and Global Change Laboratory, Agricultural Research Service, United States Department of Agriculture, Beltsville, MD, United States of America; Ospedale S. Corona, ITALY

## Abstract

Plant phenology (e.g. timing of spring green-up, flowering) is among the most sensitive indicator of ecological response to ongoing climate variability and change. While previous studies have documented changes in the timing of spring green-up and flowering across different parts of the world, empirical evidence regarding how such ongoing ecological changes impact allergic disease burden at population level is lacking. Because earlier spring green-up may increase season length for tree pollen, we hypothesized that early onset of spring (negative anomaly in start of season (SOS)) will be associated with increased hay fever burden. To test this, we first calculated a median cardinal date for SOS for each county within the contiguous US for the years 2001–2013 using phenology data from the National Aeronautics and Space Administration’s Moderate Resolution Imaging Spectroradiometer (MODIS). We categorized yearly deviations in SOS for each county from their respective long-term averages as: very early (>3 wks early), early (1–3 wks early), average (within 1 wk), late (1–3 wks late) and very late (> 3 wks late). We linked these data to 2002–2013 National Health Interview Survey data, and investigated the association between changes in SOS and hay fever prevalence using logistic regression. We observed that adults living in counties with a very early onset of SOS had a 14% higher odds of hay fever compared to the reference group, i.e. those living in counties where onset of spring was within the normal range (Odds Ratios (OR): 1.14. 95% Confidence Interval (CI): 1.03–1.27). Likewise, adults living in counties with very late onset of SOS had a 18% higher odds hay fever compared to the reference group (OR: 1.18, CI: 1.05–1.32). Our data provides the first-ever national scale assessment of the impact of changing plant phenology–linked to ongoing climate variability and change–on hay fever prevalence. Our findings are likely tied to changes in pollen dynamics, i.e early onset of spring increases the duration of exposure to tree pollen, while very late onset of spring increases the propensity of exposure because of simultaneous blooming.

## Introduction

Allergic rhinitis, commonly referred to as hay fever, affects 25 million Americans, and costs the US economy an estimated $11.2 billion/year [1;2]. It is a chronic condition characterized by itching, clear rhinorrhea, and sneezing [3–7]. Besides economic costs, hay fever also reduces quality of life and leads to missed work and school days[2;3;7;8]. The common triggers of hay fever, among others, include seasonal exposures to aeroallergens such as mold and pollen[3–6;8].

In North America, the dominant sources of pollen include trees in the spring, grass in the summer and weeds in the fall[9]. Timing of flowering of these plants is a primary determinant of pollen season length—an important determinant of exposure to allergenic pollen[10;11]. The timing of flowering is sensitive to temperature, particularly for plants that flower in early spring, such as trees.[12–15] Consequently, this important phenological event, along with the start of spring season (SOS), as well as growing season length (GSL) has undergone noticeable changes over the past several decades in response to changing climate.[12–22] For example, studies based on in situ observations have shown that the duration of the ragweed pollen season over North America has been increasing in recent decades, with the most pronounced changes observed in northern latitudes; consistent with higher rates of warming[23]. Furthermore, ragweed plants grown at an urban location showed earlier onset as well as significantly higher rates of pollen production compared to a rural location where the temperature and the CO_2_ concentration were 3.6°F and 30% lower, respectively[24]. These studies, among others, provide indirect evidence of how climate change is impacting the exposure dynamics of allergenic pollen, resulting in more severe and frequent exacerbations of allergic diseases[25]. This mechanism involving temperature, flowering phenology and pollen dynamics has also been suggested as one of the possible explanations for the observed association between exposure to extreme heat events—projected to grow in frequency, duration, and intensity in response to changing climate[26–28]—and increased risk of allergic diseases. [29;30] However, no study has directly linked the changes in plant phenology with allergic diseases or provided quantitative assessment of this association on a national scale.

Despite mounting interest in the links between climate change and adverse health outcomes[31], quantitative assessments of this association are sparse. This is due, in part, to insufficient exposure metrics that can reliably quantify, then link “exposure” to climate-induced environmental change to human health outcomes over a long temporal scale. However, in the context of hay fever, satellite based measures of floral phenology lends itself as a robust exposure metric that enables climate-induced changes to be directly linked with allergic diseases for several reasons: 1) previous studies have shown plant phenology to be a sensitive indicator of changing climate[16]; 2) floral phenology is directly related to pollen dynamics, including pollen load and pollen season length[11;16–18]; and 3) phenology data collected with identical methodology is currently available on a global scale with uniform temporal and spatial resolution.

In this study, we quantified and evaluated the association between alteration in plant phenology that have been tied with ongoing climate change and hay fever burden in the contiguous United States. We further investigated how this association varied across age, race, sex, SES status, and geographic areas.

## Materials and methods

### Exposure metric

We downloaded phenology data for the contiguous United States from the United States Geological Survey, (USGS) phenology network (http://phenology.cr.usgs.gov) at a 250 m spatial resolution. These data were derived based on the changes in weekly time series of Normalized Difference Vegetation Index (NDVI) from the National Aeronautics and Space Administration Moderate Resolution Imaging Spectroradiometer (MODIS) data as described previously[32–34]. In brief, a delayed moving average (DMA) is predicted based on previous pixel level observations, and compared to the smoothed NDVI data values. A point of departure (cardinal date) is identified when the smoothed NDVI value becomes larger than the predicted DMA value, and referred to as the start of the growing season (SOS) for that particular pixel[32–34]. We aggregated this gridded SOS data to county level by taking a median of all the pixels within a county. Because there is considerable variability in SOS within the contiguous US, we calculated a median cardinal date for onset of greening for each county using the available MODIS phenology data (2001–2013). We then calculated how yearly SOS in each county deviated from their respective long term (13 years: 2001–2013) averages and categorized these observed deviations into 5 groups: very early (>3 wks early), early (1–3 wks early), normal (within 1 wk), late (1–3 wks late) and very late (> 3 wks late). We refer to this county specific yearly deviation in SOS as alteration in plant phenology, and use it as an exposure metric in our analysis.

### Health outcome data

We obtained the health outcome data from the National Health Interview Survey (NHIS) for years 2002 through 2013. The NHIS is a cross-sectional survey of a nationally representative sample of the civilian non-institutionalized US population, which has been conducted continuously since 1957, although the survey instrument has changed over time[35]. During the study period, NHIS sampled roughly 40,000 households per year, with some households having multiple families. For each family, a sample adult was selected for a detailed interview, with a response rate ranging from 60 to 80%[36].

We identified hay fever using responses to the question: “During the past 12 months, have you been told by a doctor or other health professional that you had hay fever?” For demographic characteristics, we included age (18–34, 35–49, 50–64, 65+ years), race/ethnicity (Hispanic, non-Hispanic black, non-Hispanic white, all other races and ethnicities), sex (female, male), education level (less than high school/GED, high school/GED, some college, Bachelor’s degree, Graduate degree), and family income relative to the federal poverty threshold (less than 100%, 100% to less than 200%, 200% to less than 400%, 400% or above the poverty threshold)[37;38]. We used the NHIS multiply-imputed income data to assign poverty status level to records with missing values (percent missing ranged from 4.50% to 10.01% over 1997–2013) using NCHS-recommended methods[39]. We also included a geographical covariate to categorize each county into urban, suburban and rural categories.

### Statistical analysis

We merged the exposure metric (alteration in phenology) with the adult NHIS respondents (2002–2013) by county and survey year. Since the outcome measure is 12 month period prevalence, we used the interview month to lag exposure window to the previous 12 months and extracted the relevant exposure metric (SOS) from that temporal window. We used logistic regression to examine the association between alteration in plant phenology and self-reported hay fever after adjusting for age, race, sex and education, using SUDAAN to account for the complex clustered sample design of the NHIS.[40] Since some of the respondents (18%) lived in counties that had SOS outside the spring season (March through May), we conducted sensitivity analysis that included only those respondents who lived in the counties where the onset of greening occurred during spring season.

## Results

During 2002 through 2013, 8.1% of the respondents (N = 26,565) reported having hay fever (Table 1). Overall, slightly more female respondents reported having hay fever in the previous 12 months. Those who reported having hay fever tended to be more non-Hispanic whites, more educated, and insured. The vast majority of the study population (82%) lived in counties where the SOS (onset of greening) was detected in the spring season. Likewise, the majority of the study population (59%) lived in counties where the onset of greening occurred within 1 week of the long-term average. The distribution of respondents across the exposure variable (deviation in SOS) by survey year is depicted in S1 Table. Yearly variability in SOS across the Contiguous United States is publically available through USGS portal at https://phenology.cr.usgs.gov/get_data_250w.php.

**Table 1:** Demographic characteristics of the NHIS Respondents: 2002–2013.

Demographic Characteristics		Hay Fever Status			
		NO		YES	
		N	Weighted %	N	Weighted %
Sex					
	Male	134524	48.7	10215	42.4
	Female	167159	51.3	16350	57.6
Age					
	18–34	88560	31.7	4895	20.2
	35–49	81896	27.9	8962	35.2
	50–64	70301	23.6	8013	30.1
	> = 65	60926	16.9	4695	14.5
Race/Ethnicity					
	Non-Hispanic White	188344	70.3	18686	77.7
	Non-Hispanic Black	46566	11.9	3348	9.1
	Hispanic	50636	12.8	3191	8.6
	Other	16137	5	1340	4.7
Education					
	<High School	139008	44.4	9343	33.8
	High School	87631	29.6	8436	31.7
	College	49178	17.1	5441	21.4
	> College	25866	8.9	3345	13
Poverty					
	Poverty Ratio <1	50792	12.8	3660	10.1
	Poverty Ratio 1 to 2	63325	18.8	4675	15.1
	Poverty Ratio 2 to 4	90579	30.8	7507	28.8
	Poverty Ratio >4	96987	37.7	10723	46
Insurance Status					
	Un-Covered	53421	17	3213	11.3
	Covered	248262	83	23352	88.7
Timing of onset of greenness					
	Spring	242974	82.3	21283	81.6
	Summer	18966	6	1578	5.6
	Fall	13203	3.8	1316	4.5
	Winter	26540	7.9	2388	8.4
Changes in Plant Phenology					
	> 3 wks early	15844	4.7	1531	5.1
	>1–3 wks early	54392	18	4562	17.2
	1 wk early—1 wk late	174140	59.5	15141	59.1
	>1–3 wks later	35366	11.2	3335	11.8
	> 3 wks later	21941	6.6	1996	6.9

Non-Hispanic white adults had a 42% higher odds of reporting hay fever in the previous 12 months (OR 1.42, 95% CI 1.34–1.50) compared to Hispanic adults (Table 2). Likewise, individuals with a college education were more likely to report having hay fever in the previous 12 months compared to those who had less than a high school education (OR 1.71, 95% CI: 1.62–1.8). Individuals residing in counties where SOS arrived very early (> 3 weeks earlier than the long term (13-year) average) had a13% increased odds of hay fever (OR 1.13, 95% CI: 1.05–1.22) compared to those who lived in areas where the SOS arrival was within normal range (+/- 1 week of long term average). Similarly, individuals living in counties where SOS arrived very late (> 3 weeks later than the long term average) had a 14% increased odds of hay fever (OR 1.14, 95% CI: 1.06–1.21). When we restricted the analysis to the counties with springtime SOS (82% of study population), the effects were slightly stronger for very late SOS (OR 1.14, 95% CI: 1.06–1.21 vs OR 1.18, 95% CI 1.05–1.32 for full model and restricted model, respectively).

**Table 2:** Odds Ratios (OR) and 95% Confidence Intervals (CI) for hay fever prevalence among NHIS respondents (2002–2013).

		OR	9%% CI	OR^1^	95% CI
Changes in Plant Pnenology					
	> 3wk Early	1.13	1.05–1.22	1.14	1.03–1.27
	1–3 wk Early	0.96	0.92–1.00	0.95	0.9–1.00
	1 wk early to 1 wk late	1.00	REF	1.00	REF
	1–3 wk Late	1.05	0.99–1.11	1.05	0.99–1.12
	>3 wk Late	1.14	1.06–1.21	1.18	1.05–1.32
Age Groups					
	18–34	1.00	REF	1.00	REF
	35–49	1.91	1.82–2	1.86	1.76–1.96
	50–64	1.90	1.81–1.99	1.85	1.75–1.95
	> = 65	1.32	1.25–1.39	1.25	1.17–1.32
Race/Ethnicity					
	Non-Hispanic White	1.42	1.34–1.5	1.39	1.29–1.5
	Non-H Black	1.05	0.98–1.13	1.04	0.95–1.14
	Hispanic	1.00	REF	1.00	REF
	Other	1.14	1.04–1.26	1.11	0.99–1.25
Sex					
	Male	1.00	REF	1.00	REF
	Female	1.30	1.26–1.34	1.29	1.25–1.34
Education					
	<High School	1.00	REF	1.00	REF
	High School	1.37	1.32–1.42	1.38	1.32–1.43
	College	1.53	1.46–1.6	1.54	1.46–1.62
	> College	1.71	1.62–1.8	1.72	1.62–1.83

^1^Springtime Interview only

**Fig 1:**
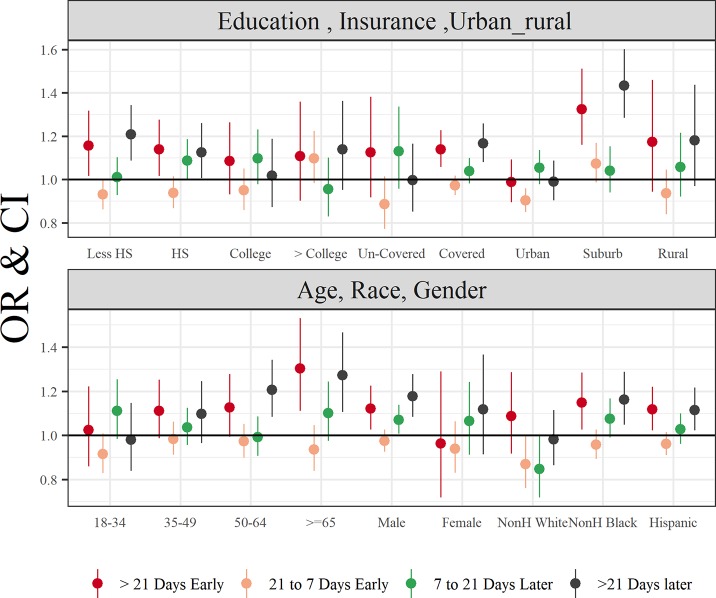
Odds Ratios and 95% CIs for changes in plant phenology and prevalence of hay fever, stratified by demographic characteristics, urban/rural status, and insurance coverage.

We stratified the analysis by demographic characteristics and urban/rural status (Fig 1). This analysis revealed a consistent U-shaped pattern of the exposure-response relationship, i.e. very early SOS as well as very late SOS were associated with an increased odds of hay fever prevalence. The relationship was most pronounced among those with less education (high school or lower), with insurance coverage, older age groups, and among those living in suburban areas. While both non-Hispanic black and Hispanic adults had increased odds of hay fever associated with both earlier and late onset of SOS, such associations were not evident among non-Hispanic white adults (Fig 1).

## Discussion

Previous studies have shown that temporal changes in plant phenology are among the most sensitive indicators of ecological response to climate change.[12–16;21;41] Here we show that such alteration in plant phenology is associated with increased prevalence of hay fever among U.S. adults. This is, to our knowledge, the first national scale assessment linking climate driven alteration in plant phenology with prevalence of hay fever in the contiguous United States.

Our findings are based on health outcomes that were derived from a nationally representative sample of the US population, and exposure metrics that were derived using satellite based observations. Our data show that very early SOS is associated with higher prevalence of hay fever. Early SOS is likely associated with earlier onset of flowering among species that bloom in the spring, such as trees[10;11;18;21]. It is plausible that earlier tree flowering may lead to longer tree pollen season, which is the dominant source of allergenic pollen during spring [42;43]. In addition, we found that very late SOS was also associated with an increased prevalence of hay fever in the contiguous US. While unexpected, this particular finding is likely related to the propensity of pollen exposure–i.e. during late SOS, most trees bloom simultaneously. It is likely that simultaneous bloom, or anthesis may result in very sharp increases in pollen concentrations, albeit for a short duration. This U-shaped exposure-response function observed suggests that there may exist a suitable window of exposure for minimal risk. Our data also suggest that changes in this particular window–both early or late—contribute to an overall increase in hay fever burden. Given over 10% of Americans are sensitized to tree pollen[44] while over 8% of US adults suffer from hay fever[36], even a modest changes in the timing of tree flowering can have a substantial impact on hay fever morbidity.

This is of particular concern among people who suffer from allergic diseases given the increasing number of studies that have shown that climate change is directly contributing to alteration in plant phenology[45]. Recent data from Europe has shown that ongoing warming has been advancing flowering by 5.2 days/decade[46], while others have reported SOS in the US is advancing by as much as 0.3 days/year.[47] The extent to which the warming climate may alter plant phenology depends upon several factors including fulfillment of the chilling requirement.[11;48–50]. If wintertime temperature is well below the chilling requirement, then the decreased winter chilling related to climatic warming does not impact the thermal time required for budburst and anthesis [11;48;49]. In such cases, warming temperature lead to earlier budburst. However, if wintertime temperature is not cold enough, vernalization may not occur, in which case plants will require larger thermal time to reactivate growth. This may result in minimal change in the timing of budburst and flowering. [11;48;49] Overall, chilling or vernalization will also need to be taken into account to improve the empirical links between pollen phenology and human health.

Overall the current study used a large nationally representative sample of non-institutionalized populations (N = 328,248) covering over a decade of survey data (2002–2013). The data were collected using identical protocols, minimizing temporal bias in outcome assessment. Our exposure metric was derived using satellite observations that incorporated the same consistent method. By using deviation in SOS from the long-term average, we controlled for the inherent variability in the timing of SOS that exists across a large geographic area. Thus, our results are associated with true alteration in plant phenology, rather than simple geographical contrasts that exist by virtue of latitude.

While the current effort represents a robust analysis, there are additional caveats that should also be considered. For example, NHIS is a multipurpose survey, and does not include more in-depth information such as date of onset of the symptoms or quantification of clinical indicators of allergen sensitization, nor does it contain information regarding allergen exposure. Furthermore, the outcome measure “hay fever” is a lay term that is often used to describe seasonal allergic rhinitis[5]. It is unclear the extent to which this term is misunderstood, and how this may vary across different ethnic groups or if the understanding has changed over time. We also did not investigate how hay fever is related to other outcomes including asthma and eczema in this study population. In addition, we used ordinal variable to categorize the SOS deviation into five groups. While the variable maintains a clear ordering of the categories, the exact distance between the categories may not be the same. Thus comparison of risk estimates across the exposure strata should be done judiciously. Likewise, we did not account for the temperature or moisture content that are related to chilling requirement and plant growth.

## Conclusion

Our results provide the first ever national-scale assessment of the association between climate change related alteration in plant phenology and hay fever among nationally representative sample of US adults. As such, they confirm a clear link between floral phenology and allergenic disease that is perturbed by climate change.

## Supporting information

S1 Table Distribution of respondents across exposure categories (SOS) by survey year. (DOCX)
