# Supplementary material for: Associations between alteration in plant phenology and hay fever prevalence among US adults: Implication for changing climate
Source: PLoS One. 2019 Mar 28;14(3):e0212010. doi: 10.1371/journal.pone.0212010 (PMC6438449; doi:10.1371/journal.pone.0212010)
Supplement: S1 Table — (DOCX) [file pone.0212010.s001.docx]

| SOS Deviation | 2002 | | 2003 | | 2004 | | 2005 | | 2006 | | 2007 | | 2008 | | 2009 | | 2010 | | 2011 | | 2012 | | 2013 | |
| --- | --- | --- | --- | --- | --- | --- | --- | --- | --- | --- | --- | --- | --- | --- | --- | --- | --- | --- | --- | --- | --- | --- | --- | --- |
|  | N | % | N | % | N | % | N | % | N | % | N | % | N | % | N | % | N | % | N | % | N | % | N | % |
| Very Early | 2342 | 7.5 | 1596 | 5.1 | 3006 | 9.6 | 1739 | 5.5 | 1457 | 6.1 | 1881 | 8.0 | 1095 | 5.0 | 2296 | 8.5 | 2689 | 9.6 | 2791 | 8.5 | 2732 | 7.9 | 2500 | 7.2 |
| Early | 1721 | 5.5 | 5279 | 17.0 | 5091 | 16.3 | 2324 | 7.4 | 1110 | 4.6 | 4812 | 20.5 | 2886 | 13.1 | 2136 | 7.9 | 11640 | 41.6 | 8538 | 25.9 | 8837 | 25.7 | 5303 | 15.3 |
| Normal | 18054 | 57.8 | 17301 | 55.6 | 18866 | 60.5 | 22637 | 72.1 | 15989 | 66.6 | 11979 | 51.2 | 13621 | 61.8 | 16617 | 61.6 | 10559 | 37.7 | 16141 | 48.9 | 12972 | 37.7 | 17153 | 49.6 |
| Late | 6172 | 19.8 | 5158 | 16.6 | 1588 | 5.1 | 2339 | 7.4 | 1986 | 8.3 | 1481 | 6.3 | 1778 | 8.1 | 4009 | 14.9 | 2154 | 7.7 | 3053 | 9.2 | 4685 | 13.6 | 4696 | 13.6 |
| Very Late | 2921 | 9.4 | 1808 | 5.8 | 2635 | 8.4 | 2373 | 7.6 | 3457 | 14.4 | 3265 | 13.9 | 2678 | 12.1 | 1933 | 7.2 | 962 | 3.4 | 2484 | 7.5 | 5148 | 15.0 | 4964 | 14.3 |
| Total | 31210 | 100 | 31142 | 100 | 31186 | 100 | 31412 | 100 | 23999 | 100 | 23418 | 100 | 22058 | 100 | 26991 | 100 | 28004 | 100 | 33007 | 100 | 34374 | 100 | 34616 | 100 |
